# Metal-backed versus all-polyethylene tibial components in primary total knee arthroplasty: A meta-analysis and systematic review of randomized controlled trials

**DOI:** 10.3109/17453674.2011.618913

**Published:** 2011-11-24

**Authors:** Tao Cheng, Guoyou Zhang, Xianlong Zhang

**Affiliations:** ^1^Department of Orthopaedic Surgery, Shanghai Sixth People's Hospital, Shanghai Jiao Tong University School of Medicine, Shanghai; ^2^Department of Hand and Plastic Surgery, Second Affiliated Hospital, Wenzhou Medical College, Wenzhou, People's Republic of China; ^3^Department of Dermatology, University Hospital Schleswig-Holstein, University of Lübeck, Lübeck, Germany

## Abstract

**Background and purpose:**

The choice of either all-polyethylene (AP) tibial components or metal-backed (MB) tibial components in total knee arthroplasty (TKA) remains controversial. We therefore performed a meta-analysis and systematic review of randomized controlled trials that have evaluated MB and AP tibial components in primary TKA.

**Methods:**

The search strategy included a computerized literature search (Medline, EMBASE, Scopus, and the Cochrane Central Register of Controlled Trials) and a manual search of major orthopedic journals. A meta-analysis and systematic review of randomized or quasi-randomized trials that compared the performance of tibial components in primary TKA was performed using a fixed or random effects model. We assessed the methodological quality of studies using Detsky quality scale.

**Results:**

9 randomized controlled trials (RCTs) published between 2000 and 2009 met the inclusion quality standards for the systematic review. The mean standardized Detsky score was 14 (SD 3). We found that the frequency of radiolucent lines in the MB group was significantly higher than that in the AP group. There were no statistically significant differences between the MB and AP tibial components regarding component positioning, knee score, knee range of motion, quality of life, and postoperative complications.

**Interpretation:**

Based on evidence obtained from this study, the AP tibial component was comparable with or better than the MB tibial component in TKA. However, high-quality RCTs are required to validate the results.

The design of the tibial component is an important factor for implant failure in total knee arthroplasty (TKA) (Pagnano et al. 1999, Forster 2003, Gioe et al. 2007b, Willie et al. 2008, Garcia et al. 2009, KAT Trial Group 2009). The metal-backed (MB) design of tibial component has become predominant in TKA because it is thought to perform better than the all-polyethylene (AP) design (Muller et al. 2006, Gioe et al. 2006, 2007a,b). In theory, the MB tibial component reduces bending strains in the stem, reduces compressive stresses in the cement and cancellous bone beneath the baseplate (especially during asymmetric loading), and distributes load more evenly across the interface (Bartel et al. 1982, 1985, Taylor et al. 1998). However, critics of the MB tibial component claim that there are expensive implant costs, reduced polyethylene thickness with the same amount of bone resection, backside wear, and increased tensile stresses at the interface during eccentric loading (Bartel et al. 1982, 1985, Pomeroy et al. 2000, Rodriguez et al. 2001, Li et al. 2002, Muller et al. 2006, Blumenfeld and Scott 2010, Gioe and Maheshwari 2010).

In the past decade, several randomized controlled trials (RCTs) have been performed to assess the effectiveness of the MB tibial component (Adalberth et al. 2000, 2001, Gioe and Bowman 2000, Norgren et al. 2004, Hyldahl et al. 2005a, b, Muller et al. 2006, Gioe et al. 2007, Bettinson et al. 2009, KAT Trial Group 2009). However, data have not been formally and systematically analyzed using quantitative methods in order to determine whether the MB tibial component is indeed optimal for patients in TKA. In this study, we wanted (1) to determine the scientific quality of published RCTs comparing the AP and MB tibial components in TKA using Detsky score (Detsky et al. 1992) and (2) to conduct a meta-analysis and systematic review of all published RCTs that have compared the effects of AP and MB tibial components on the radiographic and clinical outcomes of TKA.

## Methods

Our study conformed to the PRISMA guidelines for reporting of meta-anlyses and systematic reviews (Moher et al. 2009). We searched PubMed (1985 to February 2009), EMBASE (1988 to February 2009), Scopus (1982 to February 2009), and the Cochrane Central Register of Controlled Trials (Issue 2, 2009). We used the key words all-polyethylene, metal-backed, total knee arthroplasty, total knee replacement, TKA, and TKR to search the electronic database for RCTs that had evaluated and compared the performance of the AP and MB tibial components in primary TKA. We did not set any restrictions on language and on the duration of follow-up. However, we excluded all observational studies and case series. Furthermore, manual searching was done in the following 7 major orthopedic journals for the years 1990–2009: Journal of Bone and Joint Surgery (American and British), Clinical Orthopaedics and Related Research, Acta Orthopaedica, The Knee, Knee Surgery Sports Traumatology Arthroscopy, and The Journal of Arthroplasty. Two reviewers (TC and GZ) independently screened the titles and abstracts of identified papers, and full-text copies of all potentially relevant studies were obtained. The reference lists of the retrieved articles were also screened for any available information.

Methodological quality was independently assessed by two reviewers (TC and GZ) using the 21-point study-quality-assessment Detsky score (Detsky et al. 1992). Discrepancy regarding selection of studies was resolved by discussion with the senior author (XZ). The methodological quality of the RCT was assessed using Detsky score, which is a 14-item scoring system that contains the following domains: eligibility criteria, adequacy of randomization, description of therapies, assessment of outcomes, and statistical analysis.

The following variables were reviewed in all comparative studies, and statistically significant differences between treatment groups in the studies were noted: radiographic outcomes (alignment of the lower limb, implant placement, radiolucent line), and clinical outcomes (knee score, knee range of motion, quality of life, postoperative complications).

### Statistics

For dichotomous outcomes, risk ratio (RR) and 95% confidence limits (CIs) were calculated. Any p-values of less than 0.05 were considered statistically significant. I^2^ test for heterogeneity was conducted on the pooled results of the studies. Data from comparable studies were collated using fixed effects model unless evidence of heterogeneity across studies existed. If there were insufficient mean and standard deviation/standard error data, and meta-analysis was not possible, a systematic review was performed. Publication bias among the studies included was assessed graphically using funnel plots. The meta-analysis was conducted by one investigator (GZ) using SPSS software version 13.0 (SPCC Inc., Chicago, Illinois, USA) and RevMan software version 5.0 (Nordic Cochrane Center, Copenhagen, Denmark).

## Results

**Figure 1.**
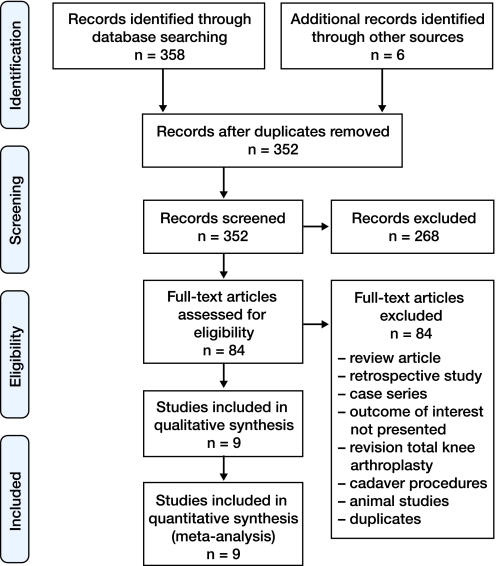
Flow diagram of selection process.

**Figure 2.**
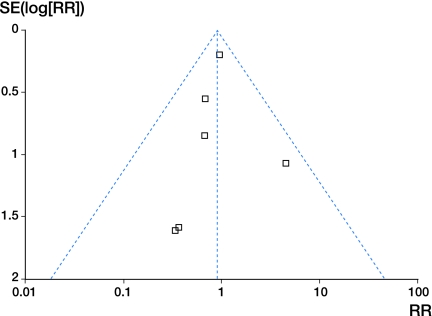
Funnel plot for these studies reporting postoperative complications after total knee arthroplasty.

In the initial search we identified 364 potentially relevant studies. After reviewing titles and abstracts and applying the inclusion and exclusion criteria, only 10 articles (Adalberth et al. 2000, 2001, Gioe and Bowman 2000, Norgren et al. 2004, Hyldahl et al. 2005a, b, Muller et al. 2006, Gioe et al. 2007, Bettinson et al. 2009, KAT Trial Group 2009) fulfilled the inclusion and exclusion criteria in the systematic review and meta-analysis (Figure 1 and Table 1). 2 of them (Gioe and Bowman 2000, Gioe et al. 2007) were reports on the same cohort at different follow-up periods. The randomization process was described and was appropriate for 5 studies (Hyldahl et al. 2005a, b, Muller et al. 2006, Bettinson et al. 2009, KAT Trial Group 2009). The authors of 4 studies mentioned randomization allocation but lacked a description of the randomization method (Adalberth et al. 2000, 2001, Gioe and Bowman 2000, Norgren et al. 2004, Gioe et al. 2007). With respect to allocation concealment, 5 studies (Adalberth et al. 2000, 2001, Norgren et al. 2004, Muller et al. 2006, Bettinson et al. 2009) were adequate and 4 (Gioe and Bowman 2000, Hyldahl et al. 2005a, b, Gioe et al. 2007, KAT Trial Group 2009) were unclear. Blinding of surgeons and patients was impossible, as showing patients their radiographs was part of routine care. The study population, inclusion/exclusion criteria, treatment interventions, follow-up time frame, and reported results were extracted and tabulated (Table 1). The sample sizes ranged from 23 to 566, with 407 men and 998 women—a total of 1,405 subjects. Within each study, there were no other differences between the treatment groups in terms of age, sex, or number of subjects, or in any other demographic information preoperatively. The duration of the follow-up assessment ranged from 2 to 10 years. The raw Detsky score for the included trials ranged from 11 to 18 points. The mean standardized score and standard deviation for the overall quality of the nine studies was 14 (SD 3). Funnel plot calculation showed substantial evidence of publication bias for the complication rate (Figure 2).

**Table 1.** Characteristics of studies included

Study	Methods	Participants	Interventions	Outcomes
Adalberth et al. (2000) Sweden	Sealed envelope	MB: 20 knees; AP: 20 knees Similar DC Inclusion criteria: primary OA (Ahlbäck grades 3–4, >60 years old, body weight <100kg) Exclusion criteria: previous ipsilateral knee surgery; inpropriate marking of the implant and bone	Brand: AGC (Anatomic Graduated Component) Cement: yes Patella: resurfacing PCL: retention Arthrotomy: MPP Identical postoperative regime	Radiographic outcome KSS score ROM Complications Follow-up:24months
Gioe et al. (2000) USA	Method of randomization, allocation concealment or blinding not described	MB: 102knees; AP: 111 knees Similar DC Inclusion criteria: OA, IA, PTA (≥60 years old) Exclusion criteria: necessitating bone grafting, modular stems or more constrained designs	Brand: PFC (Depuy) PCL: retention Cement: yes Arthrotomy: MPP 4 senior surgeons Identical postoperative regime	Radiographic outcome KSS score ROM SF-36 Complications Survivorship Follow-up: 3–5 years
Adalberth et al. (2001) Sweden	Sealed envelope	MB: 18 knees; AP: 20 knees Similar DC Inclusion criteria: primary OA (Ahlbäck grades 3–4, >50 years old, body weight <100kg) Exclusion criteria: previous ipsilateral knee surgery; inpropriate marking of the implant and bone	Brand: Freeman-Samuelson (Sulzer Orthopedics) Cement: yes (AP) Patella: resurfacing PCL: sacrifice(if necessary) 2 experienced surgeons Identical postoperative regime	Radiographic outcome KSS score ROM Complications Follow-up: 24months
Norgren et al. (2004) Sweden	Sealed envelope	MB: 11knees; AP: 12 knees Similar DC Inclusion criteria: primary OA (Ahlbäck grades 3–4, >60 years old, body weight <120kg)	Brand: Profix (Smith & Nephew) Cement: yes (cement applied at the cut proximal tibia and partly around the stem) Single surgeon The same surgical technique Identical postoperative regime	Radiographic outcome KSS score ROM Complications Follow-up: 24months
Hyldahl et al. (2005a) Sweden	Stratified randomization	MB: 16 knees; AP: 20 knees Similar DC Inclusion criteria: primary OA (Ahlbäck grades 3–4, Exclusion criteria: previous Surgery; unvisualized markers	Brand: AGC (Anatomic Graduated Component) PCL: retention Cement: yes (proximal cementing leaving the stem uncemented) Patella: no resurfacing Arthrotomy: medial 2 experienced surgeons Identical postoperative regime	Radiographic outcome HSS score Complications Follow-up:24months
Hyldahl et al. (2005b) Sweden	Stratified randomization	MB: 20 knees; AP: 20 knees Similar DC Inclusion criteria: primary OA (Ahlbäck grades 3–4, Exclusion criteria: previous Surgery; unvisualized markers	Brand: AGC (Anatomic Graduated Component) PCL: retention Cement: yes (proximal cementing with cement around the stem) Patella: no resurfacing Arthrotomy: medial 2 experienced surgeons Identical postoperative regime	Radiographic outcome HSS score Complications Follow-up: 24months
Muller et al. (2006) UK	Block randomization Sealed envelope	MB: 19 knees; AP: 21 knees Similar DC Inclusion criteria: OA, RA (>65 years old) Exclusion criteria: previous knee surgery; renal transplant; Paget's disease; metabolic bone disease, joint sepsis; steroid use; psychosocial or physical disability; bone deficiencies	Brand: PFC-Σ (Depuy) Arthrotomy: MPP 4 consultant surgeons Identical postoperative regime	Radiographic outcome OKS score SF-12 Follow-up: 24months
Gioe et al. (2007) USA	Method of randomization, allocation concealment or blinding not described	MB: 70knees; AP: 97 knees Similar DC Inclusion criteria: OA, IA, PTA (≥60 years old) Exclusion criteria: necessitating bone grafting, modular stems or more constrained designs	Brand: PFC (Depuy) PCL: retention Cement: yes Arthrotomy: MPP 4 senior surgeons Identical postoperative regime	Radiographic outcome KSS score ROM SF-36 Complications Survivorship Follow-up: 10 years
Bettinson et al. (2009) UK	Computer-generated random codes and stratified randomization Sealed envelope	MB: 304 knees; AP: 262 knees Similar DC Inclusion criteria: primary OA, RA (≥55 years old) Exclusion criteria: infection; unstable knee requiring constrained or semi-constrained prosthesis	Brand: Kinemax Plus (Stryker) PCL: retention Cement: yes Patella: no resurfacing The same surgical technique Identical postoperative regime	Complications Survivorship Follow-up: 10 years
The KAT Trial Group (2009) UK	Automated centralized telephone randomization and stratified randomization	MB: 202 knees; AP: 207 knees Similar DC Inclusion criteria: primary OA, RA	116 surgeons follow their standard practice	OKS score, SF-12 EQ-5D Complications Follow-up: 24months

AP: all-polyethylene; MB: metal-backed; DC: demographic characteristic; OA: osteoarthritis; RA: rheumatoid arthritis; PTA: posttraumatic arthritis; IA: inflammatory arthritis; PCL: posterior cruciate ligament; MPP: medial parapatellar; ROM: range of motion; KSS: Knee Society score; OKS: Oxford Knee score; HSS: Hospital for Special Surgery; SF: Short Form; EQ-SD: EuroQol SD.

7 studies (Adalberth et al. 2000, 2001, Gioe and Bowman 2000, Norgren et al. 2004, Hyldahl et al. 2005a, b, Muller et al. 2006, Gioe et al. 2007) used conventional radiographs to compare the radiographic outcomes (the alignment of the lower limb and that of the components) between the two groups. There was no statistically significant difference between the groups with regard to the femoral mechanical axis (Gioe and Bowman 2000, Gioe et al. 2007) and hip-knee-ankle angle (Norgren et al. 2004, Hyldahl et al. 2005a,b). The authors of 2 studies (Adalberth et al. 2000, 2001) reported that there was no statistically significant difference between the two groups with regard to the anatomic axis of the lower limb (coronal tibiofemoral angle). 5 studies (Adalberth et al. 2000, 2001, Gioe and Bowman 2000, Norgren et al. 2004, Muller et al. 2006, Gioe et al. 2007) found that the frontal alignment of the tibial component was not significantly different between the groups. However, in the sagittal plane the alignment of the tibial component was found to be controversial in the included studies. 3 studies (Adalberth et al. 2000, Gioe and Bowman 2000, Norgren et al. 2004, Gioe et al. 2007a) found no significant difference between the groups, whereas Adalberth et al. (2001) found that the AP components were positioned with a slightly more posterior tilt as compared to the MB components. In addition, 2 studies (Gioe and Bowman 2000, Gioe et al. 2007a) evaluated femoral coronal position, change in joint line, and patellar height. The authors reported no statistically significant difference between the groups at the latest follow-up. We pooled the results from 4 studies and found that evidence of radiolucent lines (< 2 mm) adjacent to the tibial component was 16 (10%) for the AP group and 41 (27.7%) for the MB group (RR = 2.8, CI: 1.7–4.6; p < 0.001; I^2^ = 47%).

For clinical assessment, 8 studies used the Oxford knee score (Muller et al. 2006, KAT Trial Group 2009), Knee Society knee score (Adalberth et al. 2000, 2001, Gioe and Bowman 2000, Norgren et al. 2004, Gioe et al. 2007), or Hospital for Special Surgery (HSS) score (Hyldahl et al. 2005a,b). Knee range of motion (ROM) as an outcome measure was documented in 5 studies (Adalberth et al. 2000, 2001, Gioe and Bowman 2000, Norgren et al. 2004, Muller et al. 2006, Gioe et al. 2007). All studies found that these functional outcomes were not significantly different between the groups at all follow-up time points. Quality of life was measured using 3 methods: Short Form-12, Short Form-36, or EuroQol-5D. 3 studies used Short Form-12 scores (Muller et al. 2006, KAT Trial Group 2009) or Short Form-36 (Gioe and Bowman 2000, Gioe et al. 2007), whereas only 1 study (KAT Trial Group 2009) used EuroQol-5D. These studies found no statistically significant difference in the quality of life scores between AP and MB tibial components.

**Figure 3.**
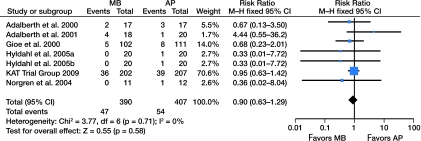
Forest plot assessing postoperative complications after total knee arthroplasty.

The authors of 7 studies provided data on postoperative complications (Adalberth et al. 2000, 2001, Gioe and Bowman 2000, Norgren et al. 2004, Hyldahl et al. 2005a, b, KAT Trial Group 2009). When we analyzed the overall complications, there were 47 in the MB group as compared to 54 in the AP group (RR = 0.9, CI = 0.6–1.3; p = 0.6, I^2^ = 0%) (Figure 3). The complications were categorized as being systemic (medical) postoperative or local (orthopedic) according to the nature of the event (Table 2). When considering both local and systematic complications, the groups were similar (RR = 0.8, CI: 0.9-1.6; p = 0.8; I^2^ = 0%; and RR = 0.9, CI: 0.6-1.5; p = 0.7; I^2^ = 0%, respectively).

**Table 2.** Summary of postoperative complications reported in the trials included in this analysis

Complications	MB	AP
Local complications		
Infection	10	14
Knee pain	0	2
Stiffness	2	3
Patellofemoral problem (patellar fracture, patellar maltracking, anterior knee pain)	2	3
Femoral neck fracture or fall	2	0
Instability	1	1
Skin complication	2	2
Surgical complications	3	0
Subtotal	22	25
Systemic complications		
DVT or pulmonary embolism	4	8
Thrombolytic complications	2	3
Myocardial infarction	1	1
Urinary complications	2	3
Medical complications	16	14
Subtotal	25	28
Grand total	47	54

DVT: deep vein thrombosis.

## Discussion

To our knowledge, this is the first systematic review and meta-analysis of RCTs comparing AP and MB tibial implants in primary TKA. Our findings show that the AP and MB tibial components gave similar radiographic and clinical results.

In our study, the frequency of radiolucent lines observed in the MB group was statistically significantly higher than that observed in the AP group. Tibial radiolucent lines may be more clearly delineated in MB components than in AP components. This may be due to the underestimated radiolucencies around the AP tibial components (Gioe and Bowman 2000). The higher incidence of radiolucent lines in the MB group may reflect this phenomenon. Non-progressive radiolucencies of 2 mm or less appear to have little clinical significance (Ritter et al. 1981, Scuderi et al. 1989, Bach et al. 2009), which corresponds to our finding that radiolucent lines had no effect on knee scores, ROM, quality of life, or postoperative complications. These results are broadly consistent with evidence from previous studies (Apel et al. 1991, Ritter et al. 1994a, b, Shen et al. 2009). In a 15-year survivorship study, Ranawat et al. (1993) reported a high incidence of tibial radiolucencies (72%), but only 2 of tibial components were loose. Although there is no direct correlation between non-progressive radiolucencies and subsequent implant loosening (Ranawat et al. 1993, Gioe et al. 2007), progressive radiolucent lines are commonly associated with early failure (Apel et al. 1991, Ritter et al. 1994a).

Metal backing of the tibial component has given lower strain and better load distribution in the proximal tibia in in-vitro biomechanical studies (Bartel et al. 1982, 1985, Small et al. 2010), which should theoretically reduce aseptic loosening and provide higher long-term survivorship of the implant. However, evidence from matched-pair or retrospective studies suggests that there is no difference in survival between AP and MB tibial components with medium-term or long-term follow-up (Apel et al. 1991, L'Insalata et al. 1992, Rand et al. 1993, Rodriguez et al. 2001, Udomkiat et al. 2001, Najibi et al. 2003, Dojcinovic et al. 2007). The findings of non-randomized cohort studies are, by nature, limited—and they are often biased due to the presence of confounding factors, including the surgeon's learning curve and patient selection. Recently, two RCTs (Gioe et al. 2007, Bettinson et al. 2009) found that survivorship, with revision for any reason as the endpoint, was similar between the two designs. Bettinson et al. (2009) reported that there was no statistically significant difference between the two designs when 10-year survivorship with aseptic failure was used as endpoint. In yet another study, Gioe et al. (2007) reported that the 10-year survivorship was marginally greater in the AP component group than in the MB component group. In fact, the overall revision rate in both groups was very low. Both groups achieved good or excellent survivorship rates for revision and reoperation after TKA during the long-term follow-up.

Although our conclusions are strengthened by the standard procedures for retrieval, assessment of relevance, and statistical processing in this systematic review and meta-analysis, a number of potential limitations should be taken into account. Firstly, there were a number of methodological limitations in the literature, including poorly randomized samples in group allocation and rare blinding of assessors or patients to the group allocation. Secondly, differences in patient population, surgical technique, outcome evaluation tool, and follow-up time may account for the clinical and statistical heterogeneity of these studies. Accordingly, the conclusions made in this review should be treated with caution. Thirdly, some variables studied in the systematic review did not shed any light on the relative role of MB or AP components, but provided a comparison between the patient groups in which the two implants were used in the studies included. For example, the early systemic complication, which implant design has little or no influence on, was associated with surgical technique and patient factors. In order to provide evidence for making the optimal choice between the two components, one should concentrate on wear rates, loosening, revision, and survivorship analysis. Finally, the consideration of cost of both tibial components could not be addressed in our analysis because none of the authors of the studies that were included reported on this subject. Although costs vary according to the brand of implant and may be determined by volume and domain contracts, AP tibial components may give cost savings (Pomeroy et al. 2000). Furthermore, none of the studies analyzed showed superiority of the MB tibial design over the AP tibial design, so we encourage use of the AP tibial component due to its low cost and excellent clinical outcomes.

In conclusion, we found similar results in the two groups in terms of knee scores, ROM, quality of life, implant alignment, and postoperative complications. Although the frequency of radiolucent lines observed in the MB group was statistically significantly higher than in the AP group, we could not prove that this corresponded to a clinically important increase in implant failure. Thus, this evidence-based literature review does not support the idea that the MB tibial component may be superior to the AP tibial component.

## References

[CIT0001] Adalberth G, Nilsson KG, Byström S, Kolstad K, Milbrink J (2000). Low-conforming all-polyethylene tibial component not inferior to metal-backed component in cemented total knee arthroplasty: prospective, randomized radiostereometric analysis study of the AGC total knee prosthesis. J Arthroplasty.

[CIT0002] Adalberth G, Nilsson KG, Byström S, Kolstad K, Milbrink J (2001). All-polyethylene versus metal-backed and stemmed tibial components in cemented total knee arthroplasty. A prospective, randomised RSA study. J Bone Joint Surg (Br).

[CIT0003] Apel DM, Tozzi JM, Dorr LD (1991). Clinical comparison of all-polyethylene and metal-backed tibial components in total knee arthroplasty. Clin Orthop.

[CIT0004] Bach CM, Mayr E, Liebensteiner M, Gstöttner M, Nogler M, Thaler M (2009). Correlation between radiographic assessment and quality of life after total knee arthroplasty. Knee.

[CIT0005] Bartel DL, Burstein AH, Santavicca EA, Insall JN (1982). Performance of the tibial component in total knee replacement. J Bone Joint Surg (Am).

[CIT0006] Bartel DL, Burstein AH, Toda MD, Edwards DL (1985). The effect of conformity and plastic thickness on contact stresses in metal-backed plastic implants. J Biomech Eng.

[CIT0007] Bettinson KA, Pinder IM, Moran CG, Weir DJ, Lingard EA (2009). All-polyethylene compared with metal-backed tibial components in total knee arthroplasty at ten years. A prospective, randomized controlled trial. J Bone Joint Surg (Am).

[CIT0008] Blumenfeld TJ, Scott RD (2010). The role of the cemented all-polyethylene tibial component in total knee replacement A 30-year patient follow-up and review of the literature. Knee.

[CIT0009] Detsky AS, Naylor CD, O'Rourke K, McGeer AJ, L'Abbé KA (1992). Incorporating variations in the quality of individual randomized trials into meta-analysis. J Clin Epidemiol.

[CIT0010] Dojcinovic S, Ait Si Selmi T, Servien E, Verdonk PC, Neyret P (2007). A comparison of all-polyethylene and metal-backed tibial components in total knee arthroplasty. Rev Chir Orthop Reparatrice Appar Mot.

[CIT0011] Forster MC (2003). Survival analysis of primary cemented total knee arthroplasty: which designs last?. J Arthroplasty.

[CIT0012] Garcia RM, Kraay MJ, Messerschmitt PJ, Goldberg VM, Rimnac CM (2009). Analysis of retrieved ultra-high-molecular-weight polyethylene tibial components from rotating-platform total knee arthroplasty. J Arthroplasty.

[CIT0013] Gioe TJ, Bowman KR (2000). A randomized comparison of all-polyethylene and metal-backed tibial components. Clin Orthop.

[CIT0014] Gioe TJ, Maheshwari AV (2010). The all-polyethylene tibial component in primary total knee arthroplasty.J Bone Joint Surg (Am).

[CIT0015] Gioe TJ, Killeen KK, Mehle S, Grimm K (2006). Implementation and application of a community total joint registry: a twelve-year history. J Bone Joint Surg (Am).

[CIT0016] Gioe TJ, Stroemer ES, Santos ER (2007a). All-polyethylene and metal-backed tibias have similar outcomes at 10 years: a randomized level I [corrected] evidence study. Clin Orthop.

[CIT0017] Gioe TJ, Sinner P, Mehle S, Ma W, Killeen KK (2007b). Excellent survival of all-polyethylene tibial components in a community joint registry. Clin Orthop.

[CIT0018] Hyldahl H, Regnér L, Carlsson L, Kärrholm J, Weidenhielm L (2005a). All-polyethylene vs. metal-backed tibial component in total knee arthroplasty-a randomized RSA study comparing early fixation of horizontally and completely cemented tibial components: part 1. Horizontally cemented components: AP better fixated than MB. Acta Orthop.

[CIT0019] Hyldahl H, Regnér L, Carlsson L, Kärrholm J, Weidenhielm L (2005b). All-polyethylene vs. metal-backed tibial component in total knee arthroplasty-a randomized RSA study comparing early fixation of horizontally and completely cemented tibial components: part 2. Completely cemented components: MB not superior to AP components. Acta Orthop.

[CIT0020] Johnston L, MacLennan G, McCormack K, Ramsay C, Walker A, KAT Trial Group (2009). The Knee Arthroplasty Trial (KAT) design features, baseline characteristics, and two-year functional outcomes after alternative approaches to knee replacement. J Bone Joint Surg (Am).

[CIT0021] Li S, Scuderi G, Furman BD, Bhattacharyya S, Schmieg JJ, Insall JN (2002). Assessment of backside wear from the analysis of 55 retrieved tibial inserts. Clin Orthop.

[CIT0022] L'Insalata JL, Stern SH, Insall JN (1992). Total knee arthroplasty in elderly patients. Comparison of tibial component designs. J Arthroplasty.

[CIT0023] Moher D, Liberati A, Tetzlaff J, Altman D G, PRISMA Group (2009). Preferred reporting items for systematic reviews and meta-analyses: the PRISMA statement. PLoS Med.

[CIT0024] Muller SD, Deehan DJ, Holland JP, Outterside SE, Kirk LM, Gregg PJ, McCaskie AW (2006). Should we reconsider all-polyethylene tibial implants in total knee replacement?. J Bone Joint Surg (Br).

[CIT0025] Najibi S, Iorio R, Surdam JW, Whang W, Appleby D, Healy WL (2003). All-polyethylene and metal-backed tibial components in total knee arthroplasty: a matched pair analysis of functional outcome. J Arthroplasty (Suppl 1).

[CIT0026] Norgren B, Dalén T, Nilsson KG (2004). All-poly tibial component better than metal-backed: a randomized RSA study. Knee.

[CIT0027] Pagnano MW, Levy BA, Berry DJ (1999). Cemented all polyethylene tibial components in patients age 75 years and older. Clin Orthop.

[CIT0028] Pomeroy DL, Schaper LA, Badenhausen WE, Suthers KE, Smith MW, Empson JA, Curry JI (2000). Results of all-polyethylene tibial components as a cost-saving technique. Clin Orthop.

[CIT0029] Ranawat CS, Flynn WF, Saddler S, Hansraj KK, Maynard MJ (1993). Long-term results of the total condylar knee arthroplasty. A 15-year survivorship study. Clin Orthop.

[CIT0030] Rand JA (1993). Comparison of metal-backed and all-polyethylene tibial components in cruciate condylar total knee arthroplasty. J Arthroplasty.

[CIT0031] Ritter MA, Gioe TJ, Stringer EA (1981). Radiolucency surrounding the posterior cruciate condylar total knee prosthetic components. Clin Orthop.

[CIT0032] Ritter MA, Herbst SA, Keating EM, Faris PM, Meding JB (1994a). Long-term survival analysis of a posterior cruciate-retaining total condylar total knee arthroplasty. Clin Orthop.

[CIT0033] Ritter MA, Herbst SA, Keating EM, Faris PM (1994b). Radiolucency at the bone-cement interface in total knee replacement. The effects of bone-surface preparation and cement technique. J Bone Joint Surg (Am).

[CIT0034] Rodriguez JA, Baez N, Rasquinha V, Ranawat CS (2001). Metal-backed and all-polyethylene tibial components in total knee replacement. Clin Orthop.

[CIT0035] Scuderi GR, Insall JN, Windsor RE, Moran MC (1989). Survivorship of cemented knee replacements. J Bone Joint Surg (Br).

[CIT0036] Shen B, Yang J, Zhou Z, Kang P, Wang L, Pei F (2009). Survivorship comparison of all-polyethylene and metal-backed tibial components in cruciate-substituting total knee arthroplasty--Chinese experience. Int Orthop.

[CIT0037] Small SR, Berend ME, Ritter MA, Buckley CA (2010). A comparison in proximal tibial strain between metal-backed and all-polyethylene anatomic graduated component total knee arthroplasty tibial components. J Arthroplasty.

[CIT0038] Taylor M, Tanner KE (1998). Freeman MA.Finite element analysis of the implanted proximal tibia: a relationship between the initial cancellous bone stresses and implant migration. J Biomech.

[CIT0039] Udomkiat P, Dorr LD, Long W (2001). Matched-pair analysis of all-polyethylene versus metal-backed tibial components. J Arthroplasty.

[CIT0040] Willie BM, Foot LJ, Prall MW, Bloebaum RD (2008). Surface damage analysis of retrieved highly crosslinked polyethylene tibial components after short-term implantation. J Biomed Mater Res B Appl Biomater.

